# Human-computer interactive physical education teaching method based on speech recognition engine technology

**DOI:** 10.3389/fpubh.2022.941083

**Published:** 2022-07-18

**Authors:** Yunpeng Sang, Xingquan Chen

**Affiliations:** ^1^Sport Department, Changshu Institute of Technology, Suzhou, China; ^2^Physical Education College, Sichuan University, Chengdu, China

**Keywords:** speech recognition, human-computer interaction, physical education, artificial intelligence, speech signal system

## Abstract

With the advent of the era of artificial intelligence, speech recognition engine technology has a profound impact on social production, life, education, and other fields. Voice interaction is the most basic and practical type of human-computer interaction. To build an intelligent and automatic physical education teaching mode, this paper combines human-computer interaction based on speech recognition technology with physical education teaching. Students input through voice signals, and the system receives signals, analyzes signals, recognizes signals, and feeds back information to students in multiple forms. For the system to process the external speech signal, this paper uses the Mel cepstral coefficient algorithm to extract the speech information. By comparing the speech recognition rate and antinoise rate of Hidden Markov Model, Probabilistic Statistics Neural Network, and Hybrid Model (Hidden Markov and Rate Statistical Neural Network combination), the speech recognition engine uses the hybrid model, and its speech recognition rate is 98.3%, and the average antinoise rate can reach 85%. By comparing the human-computer interaction physical education teaching method with the traditional teaching method, the human-computer interaction method is superior to the traditional teaching method in the acquisition of physical knowledge, the acquisition of physical skills, the satisfaction of physical education courses and the ability of active learning. It effectively solves the drawbacks of traditional physical education and rationally uses human-computer interaction technology. On the basis of not violating physical education, realize the diversification of physical education, improve the quality of teaching, improve students' individual development and students' autonomous learning ability. Therefore, the combination of human-computer interaction and physical education based on recognition engine technology is the trend of today's physical education development.

## Introduction

With the progress of the times and the vigorous development of science and technology, human-computer interaction has become the hottest topic in the field of science and technology in recent years. Human-computer interaction technology is used all time in social production, life, education, and other fields. The combination of human-computer interaction and physical education is a major research area. Human-computer interaction technology is used all time in social production, life, education, and other fields. There are many types of human-computer interaction such as image recognition, speech recognition, number recognition, and so on. Speech recognition is the most widely used in the field of human-computer interaction. Schools have always been the first places to experiment with technology. Young people in school have a strong ability to accept new knowledge. Schools have used many new technologies for a long time. For example, with the development of the Internet, the traditional teacher's blackboard teaching has long been unable to meet the needs of students to learn new knowledge. With the emergence of various multimedia, the content of teaching is rich and not boring. In some areas, there are already classroom robots to solve the current situation of lack of teacher resources and incomplete teaching content. However, the school's physical education teaching system is not perfect, there is a lack of professional physical education teachers, and students have no interest in actively participating in sports. Therefore, it is urgent to build a scientific and technological physical education system, make up for the shortcomings of traditional physical education, and integrate human-computer interaction with physical education, so that students can enjoy high-quality physical education resources.

This paper takes human-computer interaction and physical education teaching as the subject and is based on speech recognition technology. Compared with ordinary physical education teaching, this paper has the following innovations. (1) It can solve the problem of lack of physical education resources and can interact with students anytime, anywhere. (2) Highlight the subjectivity of students, students actively participate in sports, and better promote the development of students' sports. Therefore, this study is innovative.

## Related work

With the development of artificial intelligence technology and the combination of human-computer interaction and many fields, increasingly people have carried out research on human-computer interactive physical education based on speech recognition engine technology. Among them, Qian et al. adjusted the configuration relationship between the filter pooling and input feature map size to make speech recognition more stable (1). Grozdi and Jovii developed an effective method for better speech recognition by taking the difference of the acoustic properties of neutral speech and whisper, which is more stable than the traditional Mel cepstral algorithm (2). The Kim M experiment uses the maximum linear regression method to convert silent speech information into text information through the study of the speaker's lip shape (3). Darabkh et al. research pointed out that a simple and effective human-computer interaction method needs to be introduced in the education industry to reduce the cumbersome use of traditional education tools (4). Through experiments, Malallah et al. will learn that the robot receives the hand gesture instructions and makes corresponding speech according to the actions of these instructions (5). Yu et al. solved the rendering problem caused by the introduction of elastic elements on the basis of human-computer interaction technology through experiments (6). Li and Wang compared traditional school physical education teaching through experimental research and pointed out the shortcomings of traditional physical education teaching. The physical education model based on artificial intelligence technology can improve students' physical ability (7). Gaobin also pointed out the drawbacks of traditional physical education. Build a neural network model through research and use machines to recognize students' voice instructions and perform related operations according to the instructions. Through experimental analysis, he found that this human-computer interaction physical education model can better stimulate students' interest in sports (8). Through the inspiration and experimental conclusions of other researchers, the disadvantages of traditional physical education are known, and it is determined that the human-computer interaction physical education method based on speech recognition engine technology is feasible. Its disadvantage is that this kind of physical education teaching method that is out of the control of teachers may not be suitable for students who lack self-discipline or desire to learn independently.

## Human-computer interaction sports teaching method

### Speech recognition technology

**Figure 1:**
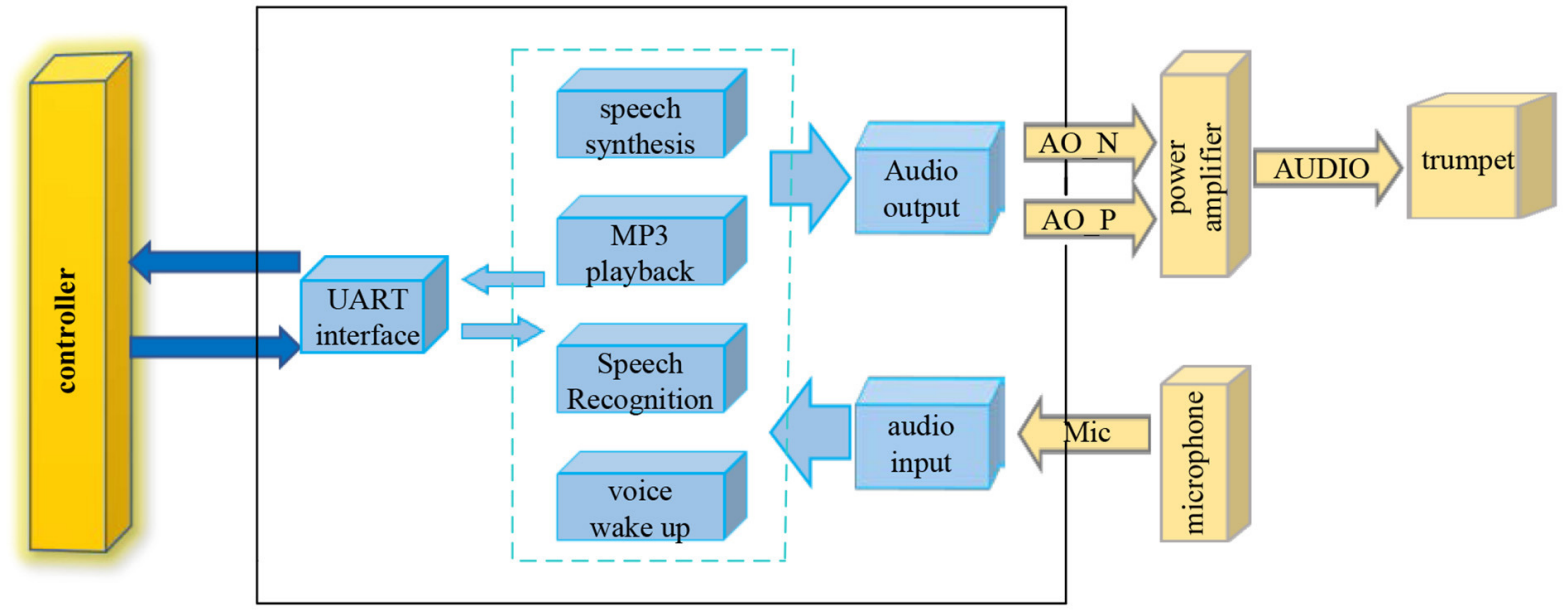
Speech recognition structure diagram.

Speech recognition technology is to convert the text content of human speech into machine language that can be understood by computers, and the computer can express it in various forms for people to understand (9). There are many ways of speech recognition, but its core architecture remains the same. Speech recognition is a pattern recognition system, and the structure of speech recognition is shown in Figure 1.

The voice transmits the unprocessed voice signal into the system through the microphone, and the system receives the voice signal and performs voice wake-up and voice recognition successively. The recognized voice signal is sent to the UART interface, and the UART interface performs feature extraction on the voice signal and identifies and matches the controller. The controller returns the voice signal with successful pattern matching to the UART interface, the voice signal is played through a voice playback device such as MP3 through voice synthesis, and finally the audio is output, and the volume of the voice can be adjusted by a power amplifier (10).

#### Voice wake-up

1) Digital conversion of voice signal

**Figure 2:**
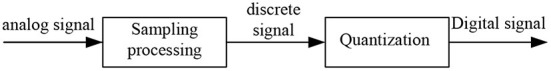
Digital conversion diagram of speech signal.

The sound is emitted from the human vocal cords in the form of analog signals. The human ear can recognize and analyze analog signals, but the computer cannot analyze them. Because computers flow information in binary form, it is necessary to convert speech signals from analog to digital (11). The basic process is generally divided into two parts: sampling processing and quantization processing. Figure 2 shows the digital conversion of the speech signal.

**Figure 3:**
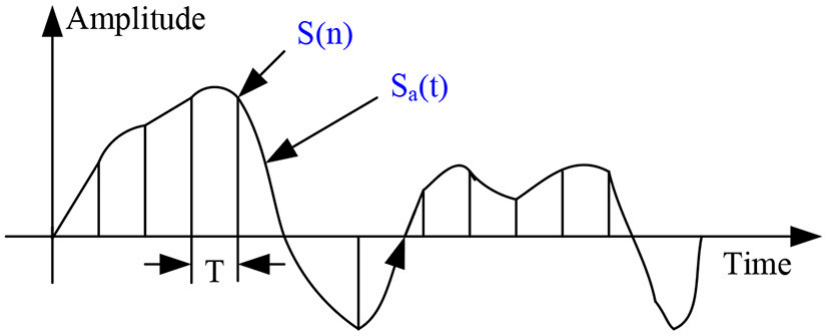
Discretization diagram of speech signal.

The process of sampling processing is to divide the analog signals sent by people at time intervals and take out a small segment of the divided segment. Each segment is called a frame. Therefore, the speech signal changes from the original curve to a frame-by-frame discrete image, and the process of sampling the originally complex and disordered analog signal is shown in Figure 3.

The mathematical expression of the discrete analog signal is as follows:


(1) $$\begin{array}{r} s\left(n\right)\;=\;s_{a}\left(nT\right)\;-\;\infty \;<\;n\;<\;+\;\infty \end{array}$$

In Formula (1), n is an integer; T is the division period, a refers to a certain curve.

Then the segmentation frequency of the speech signal is:


(2) $$\begin{array}{r} F\;=\;\frac{1}{T} \end{array}$$

Assuming that the signal width of the speech signal sa(t) is limited, when the segmentation interval is greater than twice the signal width, that is:


(3) $$\begin{array}{r} F\;=\;\frac{1}{T}\;>\;2F_{a} \end{array}$$

At this time, there will be no information bias in the sample collection information. The input voice analog signal can be restored from s(n), which means that the voice analog signal can be reconstructed through sa(t). The mathematical formula is:


(4) $$s_{a}(t)\;=\;\sum_{n=-\infty }^{+\infty }s_{a}(nT)\sin\left[\frac{\pi }{T}(t-\frac{n}{T})\right]$$

In Formula (4) *F* = 2*F*_*a*_ is the Nyquist frequency.

The quantization process is the discretization of the time division of the continuous points of the analog signal waveform.

2) High-frequency flat-lying processing

Speech is sent through the mouth and nose, and the signal has high and low frequencies. If the frequency of the signal is too high, it will be difficult to obtain the sample when it is collected. Therefore, it is necessary to flatten the high-frequency signal to increase the high-frequency recognition rate of the input speech (12).

3) Voice frame windowing

Since the voice signal is sent by people, it is unstable, and the signal fluctuation is always in a random state. The analog form of the voice signal is crooked as a whole. However, if the analog signal is divided by equal time, a small segment is divided into a frame, and the finer the division, the closer the curve of a frame is to a value (13). To reduce the slope at both ends of the speech frame, it is necessary to perform a window function processing for each frame. Let the speech frame signal be r(n) and the window function be p(n), then the signal processed by the windowing is represented by y(n), then:


(5) $$\begin{array}{r} y\left(n\right)\;=\;r\left(n\right)\;*\;p\left(n\right)\;0\leq n\leq N-1 \end{array}$$

In Formula (5), N is the number of sampling points of each speech frame.

The most widely used function expression of the window function is:


(6) $$\begin{array}{l} P(n)=0.54-0.46*\cos\frac{2\pi n}{N-1}\\ \;0\leq n\leq N-1 \end{array}$$

4) Endpoint monitoring of voice

Voice signal endpoint monitoring means that when a person sends out a voice signal, the system can accurately identify the human voice signal in the voice mixed with external noise (14).

The amplitude of the speech signal usually changes over time. Therefore, the amplitude of the described speech signal is represented by the average short-term energy. The two key factors that affect the average short-term energy are the energy and amplitude of the speech signal. The average short-term energy is mathematically defined as:


(7) $$\begin{array}{l} E_{n}\;=\;\sum_{n=-\infty }^{+\infty }{\left[s(m)p(n-m)\right]}^{2}\\ \;=\;\sum_{m\;=\;n-N+1}^{n}{\left[s(m)p(n-m)\right]}^{2}0\;\leq n\leq N-1 \end{array}$$

In Formula (7), E_n_ is the average short-term energy, p() is the window function, and s() represents the input speech signal, m represents the speech at time m.

The value of the average short-term energy E_n_ varies with time, so that the characteristics of the human input speech signal can be judged according to the value of E_n_. The average short-term energy is widely used, and it is usually used to judge the voiced and unvoiced sounds, whether it is an initial or final, and it can also be used as a special sound information.

In addition to the average short-term energy, there is an analysis standard called the zero-crossing rate in the speech signal analysis process. The simple understanding is that on the time axis, the number of times that two adjacent discrete points cross the abscissa axis, in other words, the number of times the voice curve changes between the positive and negative signs. The number of zero-crossings per unit time is called the average zero-crossings.

Therefore, the average short-term zero-crossing rate of the speech signal s(n) is mathematically defined as:


(8) $$\begin{array}{l} Q(n)\;=\;\sum_{-\infty }^{+\infty }\left|\;sgn\left[s(m)\right]-\;sgn\left[s(m-1)\right]\right|p(n-m)\\ \;=\left|\;sgn\left[s(n)\right]-\;sgn\left[s(n-1)\right]\right|*p(n) \end{array}$$

In Formula (8), the expression function of sgn[a] is:


(9) $$sgn(x)\;=\;\left\{ \begin{array}{l} 1,x\;\geq 0\\ -1,x<0 \end{array}\right.$$

The average short-term zero-crossing rate is to judge the average amplitude of changes in unit time, and its main purpose is to distinguish unvoiced and voiced sounds. It is verified by experiments that the average short-term zero-crossing rate of unvoiced sounds is relatively high, while the average short-term zero-crossing rate of voiced sounds is relatively low. Under severe noise interference, the average short-term zero-crossing rate can be used to distinguish whether there is human speech.

5) Speech signal denoising

Speech denoising is a key step in speech. Usually, the speech signal entering the system is accompanied by a lot of noise. Failure to denoise these noises will cause large deviations in speech recognition (15). The denoising of speech usually uses the spectral entropy function, and the general process of denoising is speech framing, and short-time frames are subjected to short-time Fourier transform. Therefore the probability function of the sound spectrum is:


(10) $$P_{k}=s(g_{i})÷\sum_{j=1}^{M}s(g_{j}),\;i=1,2,\cdots ,M$$

In formula (10), s(gi) represents the spectral component of the signal curve after the short-time Fourier transform, P_k_ is the probability density function, i represents the Fourier index, and M represents the length of the short-time Fourier transform.

The curve type of the speech signal is very similar to the power signal, so the power signal can be used instead of the speech signal to construct the entropy function, and the spectral entropy function curve is symmetrical. Therefore, half of it can be taken as the research object, and the Formula (10) can be modified to obtain:


(11) $$P_{k}\;=\;{\left|s(g_{i})\right|}^{2}÷\sum_{j=1}^{M/2+1}{\left|s(g_{i})\right|}^{2},\;i=1,2,\cdots ,\frac{M}{2}+1$$

The variable interpretation in Formula (11) is the same as Formula (10).

The spectral entropy can be described by the power spectrum principle. Therefore, the amplitude of the speech signal can be judged by observing the power spectrum graph, which has great practical significance for speech noise reduction. For example, when a single voice is input, the distribution range of the curve is found to be small due to the observation of the power spectrum curve. However, when the noise is input, the curve distribution has a large jumping range. Therefore, this method can be used to judge the difference between single speech and noise. Single speech can also be extracted from noise by this principle.

#### Feature extraction of speech signals

There is a essential technology in the process of speech recognition, that is, the feature extraction of speech signals. Features represent the unique points of human speech, and general speech feature parameters include resonance peak, frequency, short-term energy, and other parameters (16). The most widely used in today's technical field are the linear prediction cepstral coefficients and mel-frequency cepstral coefficients. Both methods analyze the semantics of the speech signal by converting the speech signal to the frequency spectrum and studying the characteristic parameters of the speech signal (17).

The main method of linear prediction of cepstral coefficients is linear prediction. To express clearly, the mathematical expression of the speech function after linear prediction can be set as:


(12) $$G(x)\;=\;\frac{1}{1+\sum_{i=1}^{r}a_{i}x^{-i}}$$

In Formula (12), r represents the linear order. The explanation of a is the same as Formula (1). Let g(n) be the response function, then to find g'(n) there are:


(13) $$\text{ln}G(x)\;=\;\sum_{n=1}^{+\infty }g^{\prime }(n)x^{-n}$$

Substituting Formula (12) into Formula (13), it can be got:


(14) $$\sum_{n=1}^{+\infty }ng^{\prime }(n)x^{-n+1}=\frac{\sum_{k=1}^{r}ka_{k}x^{-k+1}}{1-\sum_{k=1}^{r}a_{k}x^{-k}}$$

Simplified to get:


(15) $$\left\{ \begin{array}{l} g^{\prime }(0)=0\\ g^{\prime }(1)=a_{1}\\ g^{\prime }(n)=a_{n}+\sum_{k=1}^{n-1}\left(1-\frac{k}{n}\right)a_{k}g^{\prime }(n-k),1\leq n\leq r\\ g^{\prime }(n)=\sum_{n=1}^{r}(1-\frac{k}{n})a_{k}g^{\prime }(n-k),n>r \end{array}\right.$$

The speech features can be well-described by Formula (15).

The Mel frequency cepstral coefficient method is mainly carried out through four parts: equal time division, window function, loading, Fourier transform, and mel frequency filtering. The formula is expressed as follows:


(16) $$t_{i}=\text{ln}(\sum_{k=0}^{M-1}\left|G_{i}(k)\right|),i=1,2,\cdots ,r$$

In Formula (16), t represents the coefficients filtered by the Fourier transform.

### Hidden Markov technique

**Figure 4:**
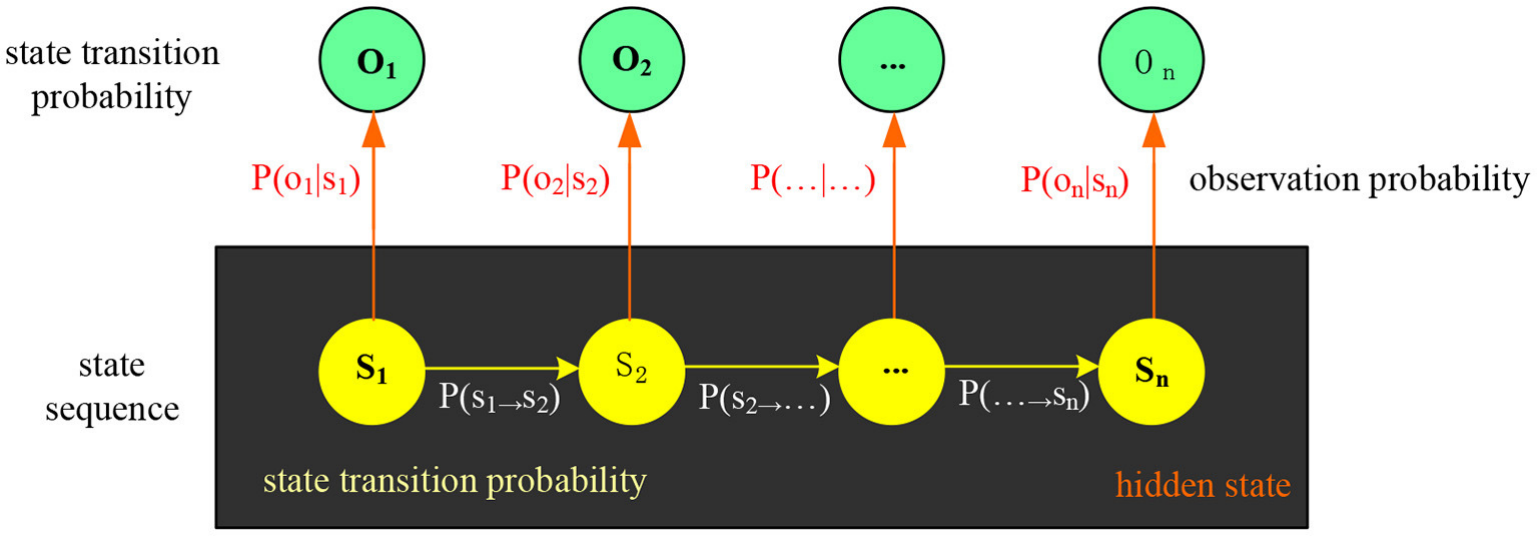
Hidden Markov Model diagram.

Hidden Markov Model is an application of probability and statistics. Hidden Markov Model has two layers of process models, namely, Markov chain and random process (18). The Markov chain is to monitor the state running in the hidden Markov model, and the random process is to associate the model state with the corresponding value, and the model state is also hidden between these corresponding values. The hidden Markov model can describe the state of the speech signal transmission process through the two-layer process model and can solve the unstable factors of the short-term signal. Figure 4 shows the hidden Markov model diagram. The application of Hidden Markov Models in the field of speech recognition is actually the process of being recognized by the ear after imitating human speech. Human ear recognition of speech is also divided into two parts. The first part is that the ear receives human speech signals, but can only hear the sound and cannot obtain semantics. The second part is the semantic analysis of the speech information and the analysis of the inner meaning of the speech signal. Speech realizes the information transfer process through these two parts, that is, the process of speech from sending to receiving and then to semantic understanding. The hidden Markov model is judged in the form of parameters. The following describes the meaning of the parameters of the hidden Markov model.

Let the total number of states in the hidden Markov model be M, but the state of the model is usually not observed, so the M states can be recorded as {*a*_1_, *a*_2_, ⋯ , *a*_*M*_}. In order to conveniently record the state information at a certain time, let u(t) be the state at time t, then there are *u*(*t*)∈{*a*_1_, *a*_2_, ⋯ , *a*_*M*_}.

Let the variable observed in each state be K. Then each state has its corresponding variable. Let K variables be recorded as *W* = {*w*_1_, *w*_2_, ⋯ , *w*_*K*_}, and set h(t) as the variable at time t, recorded as *h*(*t*)∈{*w*_1_, *w*_2_, ⋯ , *w*_*K*_}.

Set the probability distribution of state transitions to *X* = (_*x*_*ij*_)*M*×*M*_. Among them, *x*_*ij*_ indicates that the state transition probability changes from state *x*_*i*_ to *x*_*j*_ from time t to time t+1. The mathematical formula is:


(17) $$X\;=\;\left\{x_{ij}\right\},x_{ij}=P\left|x_{j}|x_{i}\right|,1\leq i,j\leq M$$

Set the probability distribution of the observed variable to *Y* = (_*y*_*jd*_)*M*×*K*_. Among them, *y*_*jd*_ represents the probability of the observed variable at time t and the state is *x*_*i*_. The mathematical formula is:


(18) $$\begin{array}{r} Y\;=\;\left\{y_{j}\left(d\right)\right\},y_{j}\left(d\right)=P|w_{d}|x_{j}|,1\leq j\leq M,1\leq d\leq K \end{array}$$

If the probability of the original state is set to f, then:


(19) $$\begin{array}{r} f\;=\;\left\{f_{j}\right\},f_{j}=P|x_{i}|,1\leq i\leq M \end{array}$$

The Hidden Markov Model is described by the above five parameters. Therefore, when these five parameters are normalized into a variable λ, the hidden Markov model can be described by λ, λ = (*M, K, X, Y, f*), where M and K are fixed numbers. Therefore the Hidden Markov Model can be expressed as λ = (*X, Y, f*).

Separating the hidden Markov model parameters yields two parts, the previously mentioned state and observed variable values. The state is determined by λ = (*X, f*), and the other part of the observed variable value is determined by Y. When a hidden Markov model parameter is known, the following observation sequence *B* = {*b*_1_, *b*_2_, ⋯ , *b*_*L*_} can be obtained. Among them, L is the total length of the observation sequence. The specific operation steps are: determine the initialization state, and find the probability of *x*_*i*_ according to λ; set the moment at this time as 1. By observing the probability distribution of *x*_*i*_, find the corresponding observed variable value b. Observing the transition probability distribution *x*_*ij*_ of *x*_*i*_, a new state can be found *x*_*j*_. At time t + 1, when t < L, we continue to return to finding the value of the observed variable bi, and the loop ends when t = L.

### Neural network technology

**Figure 5:**
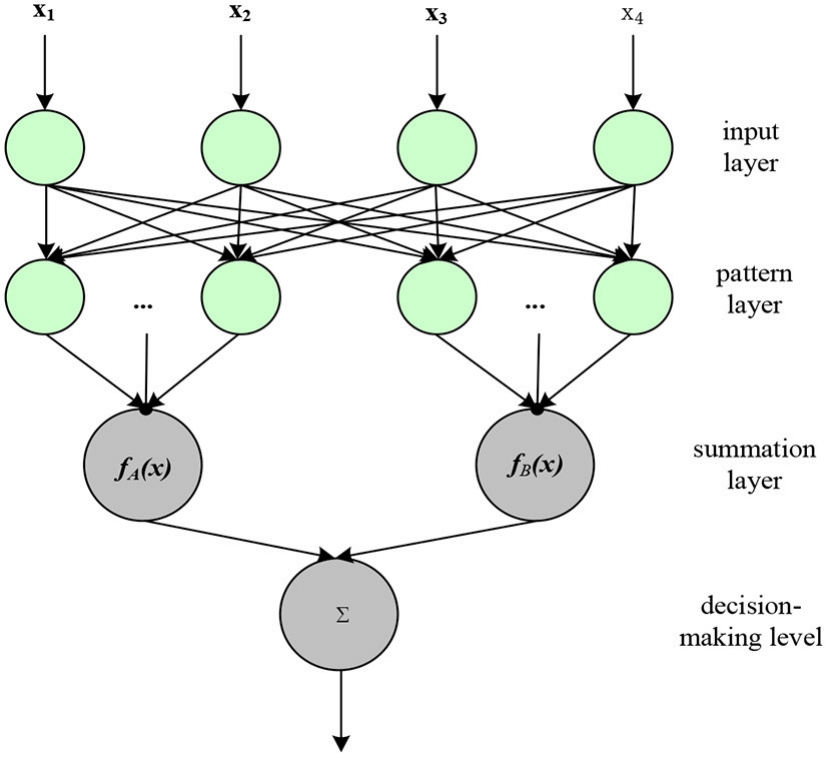
Probability and statistics neural network structure diagram.

Based on the principle of neural network technology, through continuous training of speech data, let the system learn and finally achieve a high degree of recognition of speech and semantics (19). The neural network technology used in the field of speech recognition is a probabilistic and statistical neural network. Probability and statistics neural network can recognize speech well after continuous data training through its own powerful learning ability. It is a parallel network structure based on window function (20). The structure diagram of the probability and statistics neural network is shown in Figure 5.

The probability and statistics neural network is divided into four layers. The first layer is the input layer. By receiving a large number of training sample data, the dimension of the sample determines the number of neurons. The second layer is the pattern layer, which receives the sample data (mainly the matching relationship between the feature vector and the pattern) from the input layer and calculates and analyzes it. The function formula passed from the input layer to the pattern layer is expressed as:


(20) $$\begin{array}{r} \mu \left(Z_{i}\right)\;=\;\exp{\left[\left(Z_{i}-1\right)/{\bar{\delta }}_{i}\right]}^{2},\;i=1,2,\cdots \;,d \end{array}$$

Among them, δi in Formula (20) represents the feature vector passed from the input layer to the pattern layer.

The third layer is the summation layer, which takes the output of the pattern layer as the input condition of the summation layer, and then accumulates and sums the outputs of the pattern layer. The fourth layer is the decision layer. The input of the decision layer is the output [*f*_*a*_(*x*), *f*_*b*_(*x*), ⋯ , *f*_*n*_(*x*)] from the summation layer. The number of neurons in the decision layer is related to the type of samples. The decision-making layer determines the data type of the output data, and the final output of the recognized voice information is continuously screened through the neural network.

### Voice mixed model technology

The speech hybrid model technology combines Hidden Markov Model and Probabilistic Statistics Neural Network Model. The speech mixture model combines the advantages of the above two models, which not only solves the overlapping problem of hidden Markov models, but also greatly enhances the processing of dynamic networks (21). The speech hybrid model technology can improve the accuracy of speech recognition and improve the performance of language semantic confusion.

The workflow steps of the speech hybrid model are: external speech input, preprocessing of speech information, and feature extraction for language recognition. Generally, the Mel cepstral coefficient algorithm is used, the parameter library in the hidden Markov model is used, the parameter decoding is performed, the probability and statistics neural network model is used for data training, and finally the speech is recognized. The speech hybrid model structure makes full use of the parameter system in the hidden Markov model and the self-training ability of the probability and statistics neural network model.

## Human-computer interaction physical education teaching experiment based on speech recognition engine technology

### Speech recognition experiment

#### Comparison experiment of speech recognition rate

To explore and find a human-computer interactive physical education model that is most suitable for speech recognition engine technology, it is necessary to use a model engine technology with the highest speech recognition degree as the model engine technology in this experiment (22). For this reason, this paper compares the speech recognition rate of the existing Hidden Markov Model (HMM), Probability and Statistics Neural Network Model (PNN) and Speech Mixture Model (HMM-PNN).

To verify the effectiveness of the speech recognition engine technology in this experiment, this experiment adopts the control variable method to conduct the experiment. That is, the experimental hardware equipment (sound card equipment, computer signals, etc.) used in this speech recognition rate comparison experiment must be the same. This ensures that the quality of the input voice is the same, the experimental software system chooses Windows7 system, and the programming platform uses the MATLAB platform for simulation experiments.

To ensure the real accuracy of the experiment, the sample objects of this experiment are from 1,000 students, including 500 boys and 500 girls. Voice test sample data for students to speak the specified voice vocabulary (open basketball video, close basketball video, open volleyball video, close volleyball video, what sports class is suitable for today, open, close play, pause, return). Each word is said 5 times, with a total of 50,000 speech sample data. Among them, 25,000 speech data are selected as the training sample set, and the other 25,000 speech data are used as the test sample set. The speech recognition results are shown in Table 1.

**Table 1:** Speech recognition accuracy table.

**Groups**	**Phonetic vocabulary**	**HMM**	**PNN**	**HMM-PNN**
Group 1	Open basketball video	2,370/2,500	2,300/2,500	2,450/2,500
Group 2	Close basketball video	2,450/2,500	2,330/2,500	2,490/2,500
Group 3	Open Volleyball Video	2,200/2,500	2,229/2,500	2,359/2,500
Group 4	Close volleyball video	2,390/2,500	2,410/2,500	2,480/2,500
Group 5	What sports classes are suitable for today?	2,114/2,500	2,224/2,500	2,410/2,500
Group 6	Open	2,230/2,500	2,490/2,500	2,500/2,500
Group 7	Close	2,413/2,500	2,423/2,500	2,473/2,500
Group 8	Play	2,390/2,500	2,389/2,500	2,459/2,500
Group 9	Pause	2,208/2,500	2,298/2,500	2,481/2,500
Group 10	Back	2,359/2,500	2,409/2,500	2,469/2,500
Group 11	Average accuracy	0.925	0.940	0.983

**Figure 6:**
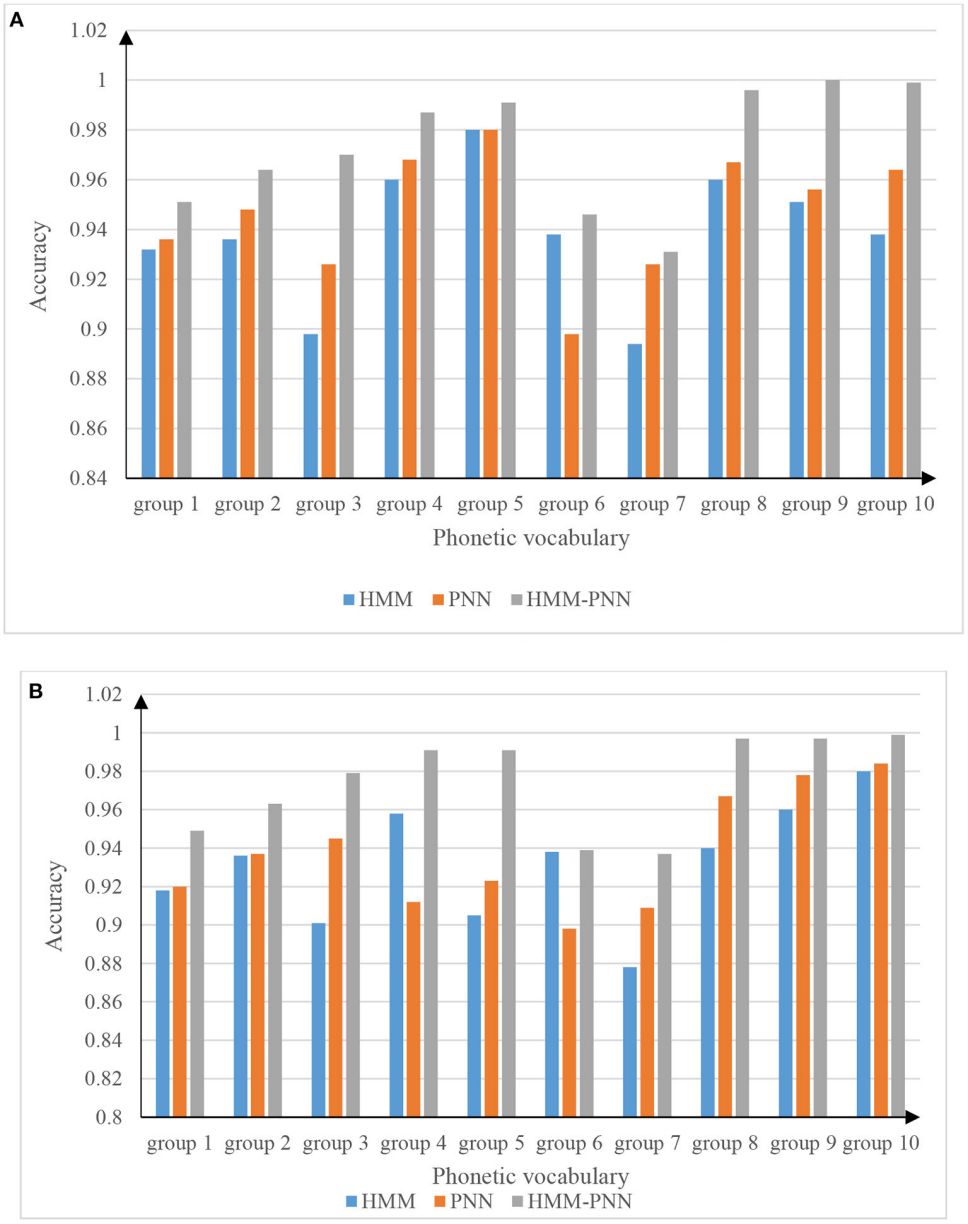
The accuracy of speech recognition for boys and girls. **(A)** Accurate picture of male voice recognition. **(B)** Accurate picture of girls' voice recognition.

In the 25,000 test samples, each word has 2,500 data, and the data in Table 1 is interpreted as, for example, 2,300/2,500 means that the test sample data is 2,500, and the number of correct identifications is 2,300. From the data in Table 1, the three models of HMM, PNN, and HMM-PNN all have high speech recognition accuracy, and the three recognition accuracy rates are all above 90%. The average recognition accuracy of HMM is 92.5%, the average recognition accuracy of PNN is 94.0%, and the average recognition accuracy of HMM-PNN is as high as 98.3%. To reflect the accuracy and validity of the experiment, the results of the test to distinguish between males and females are shown in Figure 6.

It can be seen from the table in Figure 6A that there is little difference between the recognition accuracy of the HMM model and the PNN model in the male speech recognition experiment. However, the recognition accuracy of the PNN model is slightly higher than that of the HMM model, and the HMM-PNN model has the highest speech recognition rate. In Figure 6B, although the data on the recognition accuracy is slightly different from that of boys. However, the general trend of the speech recognition accuracy of the three models is similar to that in Figure 6A. It also shows that the recognition accuracy of the HMM model and the PNN model is not much different. Compared with the first two models, the HMM-PNN model is the most accurate in speech recognition.

#### Comparison experiment of speech antinoise ratio

**Figure 7:**
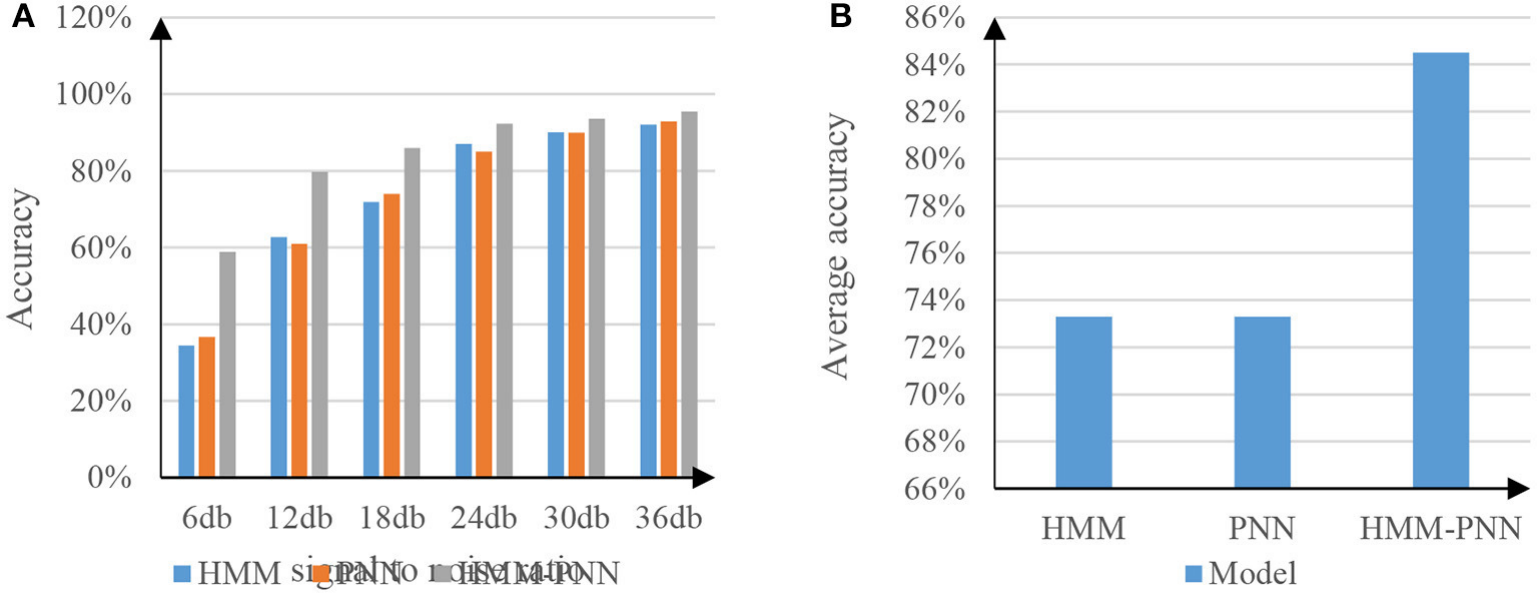
Comparison test chart of speech antinoise ratio. **(A)** The graph of the antinoise rate of the three models. **(B)** The graph of the average antinoise rate of the three models.

The speech antinoise experiment is to test the recognition depth of speech. The environment of this experiment needs to add a certain amount of signal-to-noise noise. To better distinguish the speech antinoise experiments of each model, the signal-to-noise noise added in the experiment is Gaussian noise, and the gradient of the signal-to-noise ratio is 6 db. Experiments will be carried out with SNRs of 6, 12, 18, 24, 30, and 36 db, respectively (23). The experimental results are shown in Figure 7.

The meaning of the signal-to-noise ratio is the proportion of the speech signal to the total speech. The lower the signal-to-noise ratio, the more noise is attached to the speech signal at this time (24). As can be seen from the table in Figure 7A, when the speech signal-to-noise ratio continues to increase, the accuracy of speech recognition also increases, and this rule is reflected in the above three models. Among them, the speech noise resistance rate of HMM model and PNN model is not much different, while the speech noise resistance performance of HMM-PNN model is clearly better than the former two models, and the signal-to-noise ratio is lower. The speech antinoise performance of the HMM-PNN model is more different from the first two models. Figure 7B represents the average antinoise rate results of the above three models for the entire segment with a signal-to-noise ratio of 6–36 db. The experimental results show that the average accuracy of speech recognition after full-segment antinoise for HMM model and PNN model is 73%, while the average accuracy of HMM-PNN model for full-segment antinoise speech recognition is 85%. Therefore, the HMM-PNN model is more capable of accurately recognizing speech information in noisy environments.

### Comparative experiment of traditional physical education and human-computer interaction based on speech recognition

This experiment is based on the school environment for data testing, and the human-computer interactive sports experiment is carried out on the MATLAB platform. The MATLAB platform can provide scientific computing for simulation experiments and support existing interactive technologies such as neural networks. This experiment is to explore the advantages and disadvantages of traditional physical education and human-computer interaction based on speech recognition. The speech recognition engine technology used in the experiment is the HMM-PNN model. The experiment will conduct a comprehensive evaluation from six aspects: the knowledge received by the students, the knowledge points learned, the feedback of the students, the error rate of pronunciation, the complexity of teaching, and the students' active learning ability.

The experimental samples are all from the data of different types of physical education classes of school students. To better record the data in the following experiments, the experiment is carried out using a scoring system. In the experiment, the knowledge level accepted by students is 0–100%, the knowledge points learned are 0–100%, and the feedback of students is (excellent 100%, good 75%, medium 50%, poor 25%), the speech error rate is 0–100%, and the teaching complexity is (easy 25%, normal 50%, difficult 75%, very difficult 100%), the students' active learning ability is (not active 25%, general 50%, more active 75%, very active 100%). The sample data were taken from 500 school students (300 males and 200 females). The experiment adopts the principle of the control variable method. Only way the sample students take physical education classes is changed, and the others remain unchanged. The traditional physical education teacher must also be the original teacher. To ensure the accuracy of the experiment, it is forbidden to change the physical education teacher to teach. Through the experiment, the students' learning situation of traditional physical education teaching and the physical education situation of human-computer interaction based on speech recognition are shown in Table 2.

**Table 2:** Two models of physical education teaching and learning.

**Groups**	**Physical education assessment**	**Traditional teaching**	**Human-computer interaction teaching**
Group 1	Knowledge received by students	80%	90%
Group 2	Knowledge points learned	65%	80%
Group 3	Student feedback	middle	Excellent
Group 4	Voice error rate	2%	4%
Group 5	The complexity of teaching	Difficulty	Generally
Group 6	Students' active learning ability	Not active	More active

It can be seen from Table 2 of the experimental results that the human-computer interaction physical education method has many advantages compared with the traditional physical education method. For example, it has obvious advantages in terms of knowledge received by students, knowledge points learned, feedback from students, complexity of teaching, and students' active learning ability. However, there are also shortcomings, that is, there will be some gaps between speech recognition and human ear recognition. After all, it is difficult for speech recognition engine technology to break through the recognition degree of human ears.

**Figure 8:**
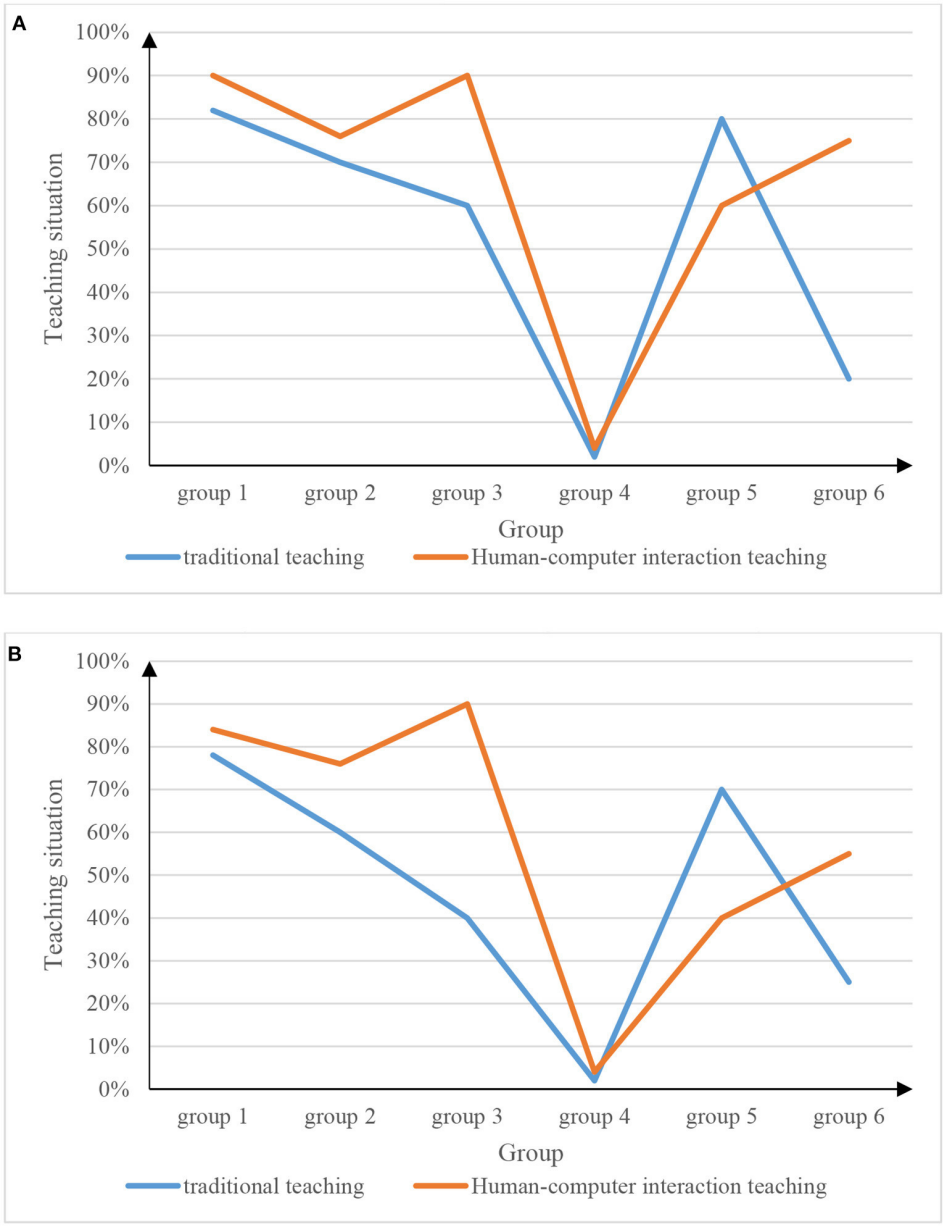
Physical education teaching situation for boys and girls. **(A)** Physical education teaching situation for boys. **(B)** Physical education teaching situation for girls.

Because there is a big distinction between men and women in physical education classes, many male students love sports and are very interested in physical education classes, but some female students do not like sports due to their own reasons, which also affects the accuracy of the experiment. To ensure the accuracy of the experiment, it is necessary to distinguish the experiments for male students and female students, and the experimental results are shown in Figure 8.

Combining Figures 8A,B, it can be got: (1) Physical education based on human-computer interaction based on speech recognition is more popular to students than traditional teaching. Students devote more energy to the teaching method of human-computer interaction and can also learn more sports knowledge. (2) Male students are generally more accepting of physical education teaching of human-computer interaction through speech recognition than female students.

### Experimental comparison of traditional physical education and human-computer interaction physical education in basketball class

The above experiment is only a general physical exercise experiment. To make the experiment more targeted, the experiment needs to conduct directional experiments on specific courses to test the actual differences between the two physical education teaching methods. The HMM-PNN model is always used in the physical education of human-computer interaction based on speech recognition, and the deviation caused by the accuracy of speech recognition and noise immunity is eliminated. The general process of human-computer interaction physical education in basketball classes is as follows: Students ask the system what physical education class is suitable for today before class, and the system will reply to students after taking into account factors such as the previous class schedule and the weather. Students can also choose courses by themselves, students can ask about teaching videos and sports precautions, students can consult the system if they do not understand through video learning, feedback their learning situation, and the system recommends course related videos, etc., or they can inquire and find by themselves.

**Figure 9:**
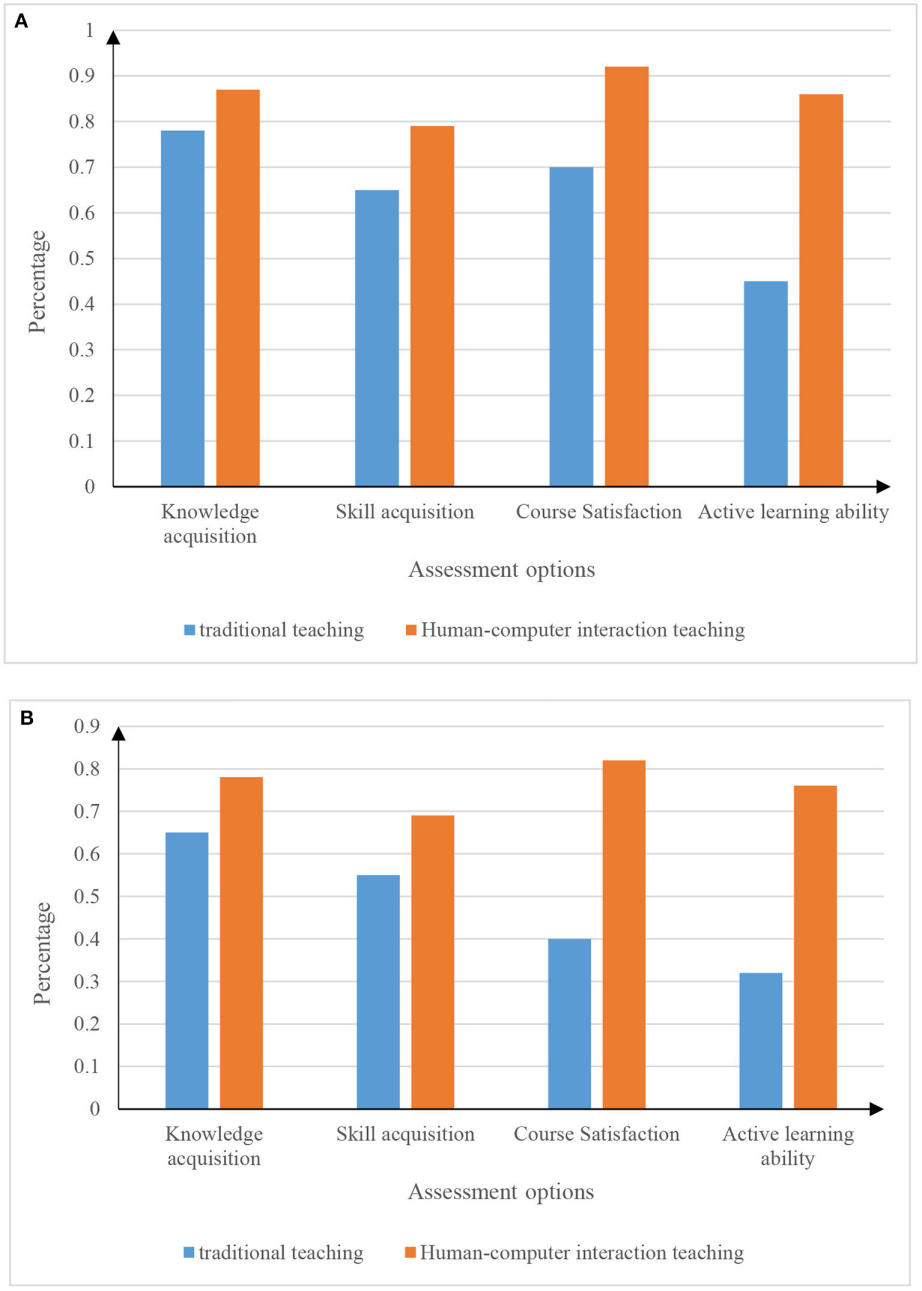
Basketball teaching situation in different grades. **(A)** Senior basketball teaching situation map. **(B)** Lower grade basketball teaching situation map.

In the experiment, 10 classes of different grades (180 students in the upper grades and 120 students in the lower grades) were selected as samples for the experiment. The sample data information is shown in Table 3. The test experiment is carried out by setting the evaluation options. The set evaluation options include basketball knowledge acquisition, basketball skill acquisition, basketball course satisfaction, and active learning ability. The experimental results are shown in Figure 9.

**Table 3:** Sample data table in basketball teaching.

**Features** **Number of people and percentage** **Grade**	**Senior grades**		**Primary level**	
	**Number of people**	**Percentage**	**Number of people**	**Percentage**
Girl	80	44.4%	56	46.7%
Boy	100	55.5%	64	53.3%
Live on campus	167	92.8%	97	80.8%
Day reading	13	7.2%	23	19.2%
Love electronics	56	31.1%	23	19.2%
Wear glasses	78	43.3%	43	35.8%
Love basketball	51	28.3%	30	25%
Experienced in human-computer interaction education	8	4.4%	3	2.5%

From the data in Figure 9, it can be seen that the physical education method of human-computer interaction is far superior to the traditional physical education method in terms of basketball knowledge acquisition, basketball skill acquisition, basketball course satisfaction and active learning ability. In particular, two aspects of basketball curriculum satisfaction and students' active learning ability. By comparing the two figures (a) and (b), we can see that the physical education teaching of human-computer interaction is slightly more affected by the senior students than the junior students.

### Experimental results

Through the speech recognition accuracy and antinoise tests, it is known that the recognition accuracy of the speech recognition model based on HMM-PNN is 98.3%, and the average antinoise rate is up to 85%.The experiment proves that the speech recognition engine technology based on the HMM-PNN model can be applied to the physical education method of human-computer interaction. Comparing the human-computer interaction physical education teaching method and the traditional physical education method, targeting groups of schools and the student as the sample experiment. Through comparative analysis in many aspects, it can be seen that the physical education of human-computer interaction is more liked by students and can greatly increase professional sports knowledge, acquire more sports skills, and improve their ability to actively learn sports knowledge.

## Discussion

Due to the vigorous development of science and technology in recent years, the school has many deficiencies in sports education, the physical quality of students has also dropped significantly, coupled with the serious shortage of professional physical education teachers and other problems, there is an urgent need for a method that can improve physical education. “Human-computer interaction +” technology has been applied to various fields such as medical care, education, service, and other fields. Therefore can the combination of human-computer interaction and physical education solve the defects of traditional physical education? Human-computer interaction can make up for the shortage of professional physical education teachers and students do not take the initiative to learn physical education knowledge. Through the experimental analysis of several speech recognition technologies, the HMM-PNN hybrid model can enable students to communicate with machines without barriers and can realize physical education for students, and can provide great help to the cause of physical education.

## Conclusions

Through the experimental study, the following conclusions are drawn from the analysis of the experimental data. (1) This paper analyzes and compares three language recognition engine technologies, namely, HMM, PNN, and HMM-PNN, and compares the recognition accuracy and noise resistance of speech recognition. The data indicates that the recognition accuracy of HMM, PNN, and HMM-PNN models are 92.5, 94.0, and 98.3%, respectively, and the noise immunity is 73, 73, and 85%, respectively. Therefore, the speech recognition engine based on the HMM-PNN model has the best effect on speech recognition. (2) By comparing the physical education method of human-computer interaction with the traditional physical education method, the human-computer interaction method is superior to the traditional teaching method in most aspects, and only slightly inferior to the traditional teaching method in speech recognition. The above two points are enough to prove that the human-computer interaction physical education teaching method based on speech recognition can be used in the field of physical education. However, completely detaching from the teacher's teaching style can be counterproductive for undisciplined students. Therefore, it is necessary to carry out projects that attract students to invest more interest in the human-computer interaction physical education system in addition to the basic physical education tasks, and to improve the recognition degree of speech recognition as much as possible. This will be the future research direction of human-computer interaction physical education teaching.

## Data Availability Statement

The original contributions presented in the study are included in the article/supplementary material, further inquiries can be directed to the corresponding author/s.

## Author Contributions

All authors have contributed to the analytic and numerical results. All authors read and approved the final manuscript.

## Conflict of interest

The authors declare that the research was conducted in the absence of any commercial or financial relationships that could be construed as a potential conflict of interest.

## Publisher's Note

All claims expressed in this article are solely those of the authors and do not necessarily represent those of their affiliated organizations, or those of the publisher, the editors and the reviewers. Any product that may be evaluated in this article, or claim that may be made by its manufacturer, is not guaranteed or endorsed by the publisher.

## Publisher's Note

All claims expressed in this article are solely those of the authors and do not necessarily represent those of their affiliated organizations, or those of the publisher, the editors and the reviewers. Any product that may be evaluated in this article, or claim that may be made by its manufacturer, is not guaranteed or endorsed by the publisher.
